# Influence of yoga on postoperative outcomes and wound healing in early operable breast cancer patients undergoing surgery

**DOI:** 10.4103/0973-6131.36795

**Published:** 2008

**Authors:** Raghavendra M Rao, H R Nagendra, Nagarathna Raghuram, C Vinay, S Chandrashekara, K. S. Gopinath, B. S. Srinath

**Affiliations:** Department of Yoga Research, Swami Vivekananda Yoga Anusandhana Samsthana, Bangalore, India; 1 Department of Clinical Immunology, M.S Ramaiah Medical Teaching Hospital, Bangalore, India; 2 Department of Surgical Oncology, Bangalore Institute of Oncology, Bangalore, India

**Keywords:** Cancer, immunity, surgery, wound healing, yoga

## Abstract

**Context:**

Pre- and postoperative distress in breast cancer patients can cause complications and delay recovery from surgery.

**Objective:**

The aim of our study was to evaluate the effects of yoga intervention on postoperative outcomes and wound healing in early operable breast cancer patients undergoing surgery.

**Methods:**

Ninety-eight recently diagnosed stage II and III breast cancer patients were recruited in a randomized controlled trial comparing the effects of a yoga program with supportive therapy and exercise rehabilitation on postoperative outcomes and wound healing following surgery. Subjects were assessed at the baseline prior to surgery and four weeks later. Sociodemographic, clinical and investigative notes were ascertained in the beginning of the study. Blood samples were collected for estimation of plasma cytokines—soluble Interleukin (IL)-2 receptor (IL-2R), tumor necrosis factor (TNF)-alpha and interferon (IFN)-gamma. Postoperative outcomes such as the duration of hospital stay and drain retention, time of suture removal and postoperative complications were ascertained. We used independent samples t test and nonparametric Mann Whitney U tests to compare groups for postoperative outcomes and plasma cytokines. Regression analysis was done to determine predictors for postoperative outcomes.

**Results:**

Sixty-nine patients contributed data to the current analysis (yoga: *n* = 33, control: *n* = 36). The results suggest a significant decrease in the duration of hospital stay (*P* = 0.003), days of drain retention (*P* = 0.001) and days for suture removal (*P* = 0.03) in the yoga group as compared to the controls. There was also a significant decrease in plasma TNF alpha levels following surgery in the yoga group (*P* < 0.001), as compared to the controls. Regression analysis on postoperative outcomes showed that the yoga intervention affected the duration of drain retention and hospital stay as well as TNF alpha levels.

**Conclusion:**

The results suggest possible benefits of yoga in reducing postoperative complications in breast cancer patients.

**After surgery, breast cancer** patients experience particularly high levels of distress[1–4] manifested as anxiety, depression and anger due to the effects of surgery and the disease itself on life expectancy, physical appearance and sexual identity.[5] Furthermore, concerns regarding one's physical condition, postoperative recovery, hospital admissions, anticipating painful procedures, image problems, confronting cancer diagnosis and worries about survival and recovery can contribute to the already prevailing distress and psychological reactions.[6] Numerous studies have shown that such preoperative distress is known to affect postoperative outcomes and delay recovery in both cancer and noncancer population.[7] In general, high preoperative stress or anxiety is predictive of greater pain intensity, longer hospital stays, more postoperative complications and poorer treatment compliance.[8,9]

Apart from this, distress is also known to impede wound healing in early phases of wound repair through its effect on glucocorticoids and proinflammatory cytokines in blood such as TNF-alpha and IL-1.[10–12] Wound healing is important in this current context of breast surgery as exaggerated inflammation, infections and collection of seroma at the wound site lengthen hospital stay, warrant more medical attention, cause distress[13,14] and lead to delayed wound closure.[15] Although the use of anesthetics and opioids for effective postoperative pain management has been shown to reduce plasma cortisol levels[16] related to poorer wound healing, they nevertheless cause distressing side effects such as headache, nausea and gastrointestinal distress and are not cost-effective.[17] Evidence suggests that in clinical practice, interventions to reduce the patient's psychological stress level may improve both wound repair and recovery following surgery.[18,19]

There is evidence to show that interventions that alter appraisal, coping and/or mood may also modulate immune and endocrine function, thereby enhancing surgical recovery.[20,21] Even modest interventions that have relatively small consequences for psychological distress such as educating the patient about surgery[18] and improving the ambience in the wards[22] are known to influence the recovery process. Several meta-analyses of presurgical intervention studies have argued that association between presurgical intervention and clinical outcome is clinically meaningful.[19] Depending on the meta-analysis, two thirds to three quarters of intervention patients had better outcomes than control subjects (patients who have undergone surgery but not intervention) with the size of improvement ranging from 20–28%.[19] These stress reduction and behavioral interventions apart from reducing distress, hospital stays and medication are also known to affect recovery from surgery and are cost-effective.[18,19,23]

Yoga is one such psychotherapeutic intervention that has been used in numerous health care concerns where stress was believed to play a role. It consists of a series of breathing exercises or *Pranayama* (regulated nostril breathing), postures, relaxation and meditative techniques. These techniques are known to alter certain physiological functions that are known to reduce the effects of stress. These functional alterations include bringing about a stable autonomic balance,[24] improvement of physical efficiency,[25] increase in cardiopulmonary functions,[26] improved immunological tolerance,[27] improved neuro-endocronine functions,[28–30] improved mood states[31–33] and a tranquil state of mind to combat stress.[34] This could be particularly useful in the current context where apart from reducing psychological distress, yoga could be used to alter endocrine and immune function to lower the risk of infections and enhance wound healing. Distress could also impede recovery by reducing compliance, for example, it is known that breathing exercises reduce the risk of pneumonia following surgery[35] and incorporating yoga interventions that use breathing, stretching and relaxation interventions could help hasten the recovery process following breast surgery. We hypothesize that yoga interventions would help improve postoperative outcomes and recovery and improve wound healing in early operable breast cancer patients undergoing surgery.

## SUBJECTS AND METHODS

Described here is a single center, randomized controlled trial that recruited ninety-eight recently diagnosed women with stage II and III operable breast cancer who were awaiting breast cancer surgery. This study evaluated the effects of yoga intervention vs a supportive therapy with exercise rehabilitation in early stage II and III breast cancer patients undergoing breast cancer surgery. The selected women were recently diagnosed with breast cancer, with time following diagnosis ranging from 1–4 weeks. Subjects were recruited from January 2000 to June 2004 at a comprehensive cancer care center in Bangalore. The study was approved by the ethical committee of the recruiting cancer center. Patients were included if they met the following criteria: i) women with recently diagnosed operable breast cancer, ii) age between 30 to 70 years, iii) Zubrod's, performance status 0–2 (ambulatory > 50% of time), iv) high school education, iv) willingness to participate, v) Surgery as a primary treatment. Patients were excluded if they had i) a concurrent medical condition likely to interfere with the treatment, ii) any major psychiatric, neurological illness or autoimmune disorders, iii) secondary malignancy, iv) presented with infections or history of recent infections in the past month. The details of the study were explained to the participants and their informed consent was obtained.

Baseline assessments were done on 98 patients on the day prior to their surgery. Sixty-nine patients contributed data to the current analyses at the second assessment (four weeks after surgery). The reasons for dropouts were attributed to migration to other hospitals, use of other complementary therapies (e.g., Homeopathy or Ayurveda), lack of interest, time constraints and other concurrent illness.

Demographic information, medical history, clinical data, intake of medications and investigative notes were taken during their initial hospital visit before randomization. About 12 ml of blood sample was collected in vacuettes under sterile conditions on the day of the surgery. Blood samples were collected between 7 a.m. to 11 a.m. for all participants to reduce diurnal variability. Follow-up assessments were done at four weeks following surgery and before the commencement of any adjuvant treatment.

### Randomization

Subjects consenting to participate in this study were randomly allocated to receive either yoga (intervention) or supportive therapy plus exercise therapy prior to their surgery using random numbers generated by a random number table. Randomization was performed using opaque envelopes with group assignments, which were opened sequentially in the order of assignment during recruitment. These envelopes had names and registration numbers written on their covers. It was not possible to mask the yoga intervention from the subjects as yoga is a popular practice. However, the investigators (treating surgical oncologists) were blind to the intervention.

### Measures

#### Postoperative outcome measures

Breast cancer subjects in our study underwent surgery as a primary treatment. They underwent either breast conservation surgery (lumpectomy with axillary dissection) or (mastectomy with axillary dissection). We assessed the following postoperative outcomes: i) Number of days of drain retention following surgery—this is indicative of seroma collection at the wound site and is known to delay wound healing.[14] The criteria for drain removal followed in the hospital was drain fluid < 50 ml in 48 hours for all breast cancer surgery patients. ii) Duration of hospital stay (number of days in hospitalization) —patients were discharged if they were ambulatory, their general condition was good and did not have any postoperative complications. iii) Postoperative duration (interval between surgery and the start of any other adjuvant treatment). iv) Interval for suture removal (number of days from the day of surgery to the day of suture removal)—the suture was removed when the approximated margins of the wound were closed (when primary union was facilitated). v) Presence of postoperative complications such as infections, secondary suturing, seroma, discharge, uncontrollable pain etc.

#### Immune outcome measures

i) Plasma levels of cytokines — TNF-alpha, IFN-gamma and soluble IL-2R alpha.

Quantifications of cytokines — sIL-2R, TNF-alpha and IFN-gamma: Two milliliters of blood samples were collected in sodium citrate vacuettes and plasma isolated for cytokine measurements.

Plasma was analyzed for cytokines using double sandwich ELISA techniques with Duoset ELISA Development kits from R & D Systems, USA. The test samples were run in duplicate and readings taken on a microplate reader (Organon Technica, USA). The test was calibrated using varying concentrations of a set of standards given along with the kit. The plates were read at 450 nm and standard curves plotted for each run with the log of cytokine concentrations on the y-axis and the log of the optical density (O.D.) readings on the x-axis; the best fit lines were determined by regression analyses. The concentrations were then extrapolated by using the mean O.D readings of the duplicate wells. The sensitivity of the tests was in the range of 15.7–998 15.8–950 and 31.5–2000 pg/ml for TNF-alpha, IFN-gamma and sIL-2 Rα' respectively.

#### Interventions

The intervention group underwent an “integrated yoga program” and the control group received “supportive counseling and postoperative exercise rehabilitation.” While the goals of yoga intervention were stress reduction and improvement in shoulder mobility, the goals of the control intervention was to reinforce social support and prevent shoulder restriction. The yoga intervention consisted of a set of breathing exercises or *Pranayama* (voluntarily regulated nostril breathing) and yogic relaxation techniques. These practices were based on the principles of attention diversion and relaxation to cope with day-to-day stressful experiences.

Supportive counseling sessions as control intervention included two important components: “i) education and reinforcing social support and ii) shoulder exercise for postoperative rehabilitation”. We used this as a control intervention mainly because it has been used earlier to hasten recovery from surgery[18,19] and to control for the nonspecific effects of the yoga program that may be associated with adjustment such as attention, support and a sense of control.

Subjects and their caretakers were invited to participate in an introductory session lasting 60 minutes before surgery where they were given information about surgery and the management of its related side effects, taught shoulder exercises and mobilization by the physiotherapist and provided the answers to a variety of common questions. Both the interventions were imparted at the patient's bedside by trained personnel during the pre- and postoperative periods and subjects underwent four such in-person sessions in the hospital. Following their discharge, subjects were asked to practise their respective interventions at home daily (for half an hour) during the next three weeks. Subjects' interventions were monitored on a weekly basis by telephone calls. Subjects were also provided audiotapes of these practices for home practice using an instructor's voice so that a familiar voice could be heard on the cassette. Subjects were also encouraged to maintain a daily log listing the yoga practices done, use of audio-visual aids for practice, duration of practice, experience of distressful symptoms, intake of medication and diet history.

### Data analysis

Data was analyzed using SPSS 10.0 for Windows. Data was tested for normality and homogeneity. An independent samples t test was done to assess postoperative outcomes between groups. The values of TNF-alpha were not normally distributed, hence, a nonparametric Mann Whitney U test was done on the change scores (pre- and postsurgery) to compare groups. A Chi Square test was done to analyze the difference in proportions among category variables across groups.

A multiple hierarchical regression analysis was done to examine the variance in dependent variables explained by independent variables. The dependent variables in this analysis were the number of days of drain retention, interval for suture removal, duration of hospital stay, postoperative duration (interval between surgery and adjuvant therapy). The independent prognostic variables entered into the analysis were age in years, type of surgery, size of tumor, postoperative surgery complications (presence or absence) and intervention. All models used the same set of five independent variables except for postsurgery TNF-alpha levels where a presurgery TNF-alpha level was added as an additional predictor. The ratio of subjects to the number of variables was 13.8 in the final model. For each equation, the probability of F-to-enter an independent variable was set at *P* < 0.10 and F- to-remove was set at *P* < 0.25. Probabilities were set at these levels so as to allow only for a small number of potential strong predictors to be selected in the models. The regression analysis was done using the entry method.

## RESULTS

The age, stages of disease, grade and node status were similar in the yoga and supportive therapy (control) groups. The Chi square test on all sociodemographic and medical characteristics of study sample did not show any significant differences in the proportions across groups [Table 1]. The mean years of education of the study sample was 12.49 ± 2.67 years with a minimum of seven years and a maximum of 17 years of education. The mean overall age of the subjects was 49.2 ± 9.6 years in both groups.

**Table 1:** Demographic characteristics

	All Subjects		Yoga group		Control group		Chi Square and (Chi square significance value)

	*n*	(%)	*n*	(%)	*n*	(%)	
Stage of Breast Cancer							
II	31	45	17	54.8	14	45.2	1.1 (3.84)
III	38	55	16	42.1	22	57.9	
Grade of Breast Cancer							
I	1	1	1	100	0	0	3.94 (5.99)
II	8	12	6	75	2	25	
III	60	87	26	43	34	57	
Menopausal status							
Pre	33	48	20	61	13	39	6.03 (7.82)
Post	33	48	11	33	22	67	
Peri	1	1	1	100	0	0	
postHysterectomy	2	3	1	50	1	50	
Type of surgery							
Mastectomy	52	75.4	24	53.8	28	46.2	0.24 (3.84)
Breast conservation	17	24.6	9	47.1	8	52.9	
Stressful life events since past 2 years							
Yes	19	28	8	42	11	58	0.34 (3.84)
No	50	72	25	50	25	50	

Control group: supportive therapy group.

Chi square significance values at *P* < 0.05 level.

### Effects of group on postoperative outcomes

Independent samples ‘t’ test was done to assess the effects of groups on postoperative outcomes. There was a decrease in the number of days of drain retention (95% confidence interval, CI (0.74 to 2.8)) following surgery. There was a rapid healing of the surgery wound as evidenced by shorter intervals for suture removal (95% CI (0.23 to 4.6)) and a decrease in the duration of hospital stay (95% CI (0.44 to 2.1)) following surgery in the yoga group as compared to controls [Table 2].

**Table 2:** Independent samples t test to compare postoperative outcomes between yoga and control groups

Postoperative outcomes	Control group	Yoga group	t value (df)	*P* value
Number of days of drain retention	6.44 ± 2.5	4.7 ± 1.6	3.46 (67)	0.001
Postoperative duration (in days)	24.6 ± 10.9	21.7±9.4	1.16 (62.7)	0.25
Interval for suture removal (in days)	12.7 ± 5.2	10.3 ± 3.6	2.21 (60.2)	0.031
Duration of Hospital stay (in days)	6.2 ± 2.1	4.9 ± 1.2	3.07 (67)	0.003

Effects of groups on postoperative outcomes in subjects undergoing only mastectomy:

Independent samples ‘t’ test was done to assess the effects of groups on postoperative outcomes. There was a decrease in the number of days of drain retention (95% CI (0.8 to 3.1)) following surgery. There was a rapid healing of the surgery wound as evidenced by shorter intervals for suture removal (95% CI (0.23 to 5.5)) and a decrease in the duration of hospital stay (95% CI (0.32 to 2.2)) following mastectomy in the yoga group as compared to controls [Table 3].

**Table 3:** Independent samples t test to compare postoperative outcomes between yoga and control groups in subjects receiving only mastectomy

Postoperative outcomes	Control group	Yoga group	t value (df)	*P* value
Number of days of drain retention	6.79 ± 2.4	4.8 ± 1.5	3.5 (45.9)	0.001
Postoperative duration (in days)	24.6 ± 10.9	21.7±9.4	0.24 (48)	0.81
Interval for suture removal (in days)	13.7 ± 5.3	10.8 ± 3.3	2.19 (48)	0.03
Duration of hospital stay (in days)	6.4 ± 2.1	5.1 ± 1.2	2.7 (44.5)	0.01

### Effects of type of surgery on postoperative outcomes

As subjects in both groups received either breast conservation surgery or mastectomy, we evaluated the effects of these two surgical procedures on postoperative outcomes. Independent samples ‘t’ test done to assess the effects of type of surgery on postoperative outcomes showed significant decreases in the interval for suture removal (95% CI (0.78 to 5.4)) and the duration of hospital stay (95% CI (0.09 to 1.9)) in subjects who underwent breast conservation surgery as compared to mastectomy [Table 4].

**Table 4:** Independent samples t test to compare postoperative outcomes between types of surgery (Mastectomy vs Breast conservation surgery)

Postoperative outcomes	Mastectomy group	Breast conservation group	t value (df)	*P* value
Number of days of drain retention	5.88 ± 2.3	4.8 ± 2.1	1.81 (67)	0.076
Postoperative duration (in days)	23.7 ± 10.1	21.2 ± 10.5	0.87 (64)	0.39
Interval for suture removal (in days)	12.4 ± 4.7	9.3 ± 3.8	2.46 (65)	0.017
Duration of hospital stay (in days)	5.8 ± 1.9	4.8 ± 1.5	1.997 (67)	0.05

### Postsurgery complications

Postsurgery complications were assessed in both the groups based on consultants' notes (blind to intervention) during the postoperative follow-up period. The presence or absence of such complications following surgery in each subject was noted as a category variable. Although there was a decrease in the number of postoperative complications in the yoga group, the independent samples' nonparametric Chi square test did not show any significant difference in the proportion of presence/absence of postoperative complications across the groups. There was also no significant difference in the postoperative complications between breast conservation vs mastectomy [Table 5].

**Table 5:** Comparison of postoperative complications between groups and types of surgeries using non-parametric Chi Square test

Postsurgery complications	Yoga, *n* = 33 *n* (%)	Control *n* = 36 *n* (%)	Mastectomy *n* = 52 *n* (%)	Breast conservation *n* =17 *n* (%)
Yes	2 (6.1)	8 (22.2)	9 (17.3)	1 (5.9)
No	31 (93.9)	27 (75)	42 (80.8)	16 (94.1)
Observed Chi Square value	3.82		1.41	
Chi square values of significance at *P < 0.05 level*	3.84		3.84	

### Cytokines

There was no significant change in soluble IL-2r alpha and IFN-gamma levels following surgery between the groups (results not shown). Wicoxon Signed rank test showed a significant increase in the TNF-alpha levels following surgery in the control group alone (*P* = 0.049). There was a significant decrease in TNF-alpha following surgery in the yoga group (*P* < 0.001) as compared to the controls in the Mann Whitney U test indicative of better wound healing in the yoga group at four weeks following surgery [Table 6].

**Table 6:** Comparison of mean scores and changes in plasma TNF-alpha levels in yoga and control groups before and after surgery

	TNF-alpha levels (pg/ml)			

Outcome measures	Pre surgery	Post surgery	Change pre-to post-	Z scores on Wilcoxons test
Control, *n* = 36				
Mean ± S.D	18.5 ±52.1	26 ± 53.5*	-7.5± 14.9	-1.97 a*
Yoga, *n* = 33				
Mean ± S.D	11.7±13.3	8.5±7.2	3.2±10.1	-0.914
Z scores for Mann Whitney Test	–	–	-3.52	–
†*P* value (Two tailed)	–	–	< 0.001	–

* *P* < 0.05, *P* value for non parametric Wilcoxons Signed rank test

† *P* value for non parametric Mann Whitney U test.

### Regression on postoperative outcomes

A multiple hierarchical regression analysis was done to examine the variance in dependent variables (postoperative outcomes) explained by independent variables such as age in years, type of surgery, size of tumor, postoperative surgery complications (presence or absence), presurgery TNF-alpha levels (for postsurgery TNF-alpha as a dependent variable only) and intervention. Number of days of drain retention was explained by the combined effects of yoga intervention (95% CI (0.32 to 2.4)), presence of surgery complications (95% CI (0.001 to 2.93)) that was responsible for 27.4% of the variance in the model. The interval for suture removal was explained by the combined effects of surgery complications (95% CI (2.8 to 8.6)) and the type of surgery (95% CI (0.37 to 5.6)) that accounted for 33.8% of the variance in the model. Duration of hospital stay was explained by intervention alone (95% CI (0.21 to 1.9)) and accounted for 21.1% of the variance in the model. None of the predictors explained variance in the postoperative duration. Lastly, postsurgery TNF-alpha levels were explained by the combined effect of presurgery TNF-alpha levels (95% CI (0.63 to 0.72)), intervention (95% CI (9.7 to 20.4)), age (95% CI (0.14 to 0.74)) and the type of surgery (95% CI (0.46 to 13.5)) that predicted 94.2% variance in the model [Table 7].

**Table 7:** Regression analysis on postoperative outcomes using predictors: age in years, type of surgery, size of tumor, postsurgery complications and presurgery plasma TNF-alpha levels

Model for	Predictor variables	R^2^	Beta	F	*P* value
Number of days of drain retention	Intervention^a^		0.298		
	Surgery a complications	0.27	0.232	4.59	0.001
Interval for suture removal	Surgery^a^		0.44		
	complications Size	0.33	0.27	6.12	< 0.001
Duration of hospital stay	Intervention^a^	0.21	0.296	3.25	0.01
Postoperative duration	–	0.09	0.245	1.287	0.282
Postsurgery
TNF-alpha levels	Presurgery TNF-alpha levels		0.963		
	Intervention^a^		0.191		
	Age in years	0.942	0.107	152.6	< 0.001
	Type of surgery^a^		0.077		

a - Category variables with two levels.

## DISCUSSION

The results suggest that yoga intervention helped reduce the length of hospital stay and improved wound healing by shortening the interval period for suture removal and reduced plasma TNF-alpha levels significantly as compared to the support and exercise intervention group following surgery. The results did not differ when postoperative outcomes were analyzed in only the mastectomy group. Regression analysis on postoperative outcomes showed intervention to affect the duration of drain retention and hospital stay and TNF-alpha levels. The interval for suture removal was explained by surgery complications and the type of surgery.

Sustained elevated levels of TNF-alpha at four weeks following surgery is indicative of stress and delayed wound healing. This could be attributed to stress-induced increase in cortisol levels in the pre- and immediate postoperative period damping inflammation or infections in the postoperative period.[36] The results showed that our intervention was effective in reducing postoperative complications. The type of surgery (mastectomy vs breast conservation) did not affect postoperative outcomes in our study which is consistent with earlier findings.[37]

In our study, there was a decrease of TNF-alpha levels following four weeks of surgery in the intervention group indicating that our intervention helped reduce risk factors in the pre- / postoperative period that could have delayed wound healing. However, an earlier study with relaxation training for breast cancer patients undergoing radiotherapy did not show any significant reductions in TNF-alpha levels.[38] This could be because radiation therapy has been shown to induce proinflammatory cytokine levels in earlier studies irrespective of stress levels possibly confounding the effects of stress reduction intervention.

Stress response can have an impact on wound healing as it is regulated by inflammatory mediators. Depression and stress are associated with enhanced production of proinflammatory cytokines.[39] Results from animal models show that stress stimulates proinflammatory genes, delays wound contraction and myofibroblast differentiation leading to delayed wound closure by > 25% and decreases immune / inflammatory responses required for bacterial clearance leading to infection in the host.[40–42] Given the substantial contribution of stress for wound repair and immune changes associated with distress, even small alterations in anxiety could have substantial clinical implications both directly through physiological mechanisms and indirectly through increased pain and decreased compliance.[7,43]

Our findings are consistent with earlier studies using behavioral and relaxation approaches to improve postoperative outcomes. A variety of hypnotic-relaxation interventions appear to shorten hospital stays, decrease pain and promote faster recovery following surgery.[44] For example, 241 patients undergoing a stressful medical procedure were randomized to receive perioperative standard care, structured attention or self-hypnotic relaxation. The self-hypnotic relaxation patients showed lower pain and anxiety, lower use of self-administered pain medication, shorter procedure times and less hemodynamic instability than the other two groups.[45] Others such as relaxation with guided imagery and exercise have demonstrated stress-relieving outcomes closely associated with wound healing.[46,47]

We propose several mechanisms for action for our yoga intervention. The internal awareness and relaxation associated with these practices are known to alter perceptible thoughts and emotions and reduce reactivity to stressful situations or stimuli thereby altering stress responses and reducing distress.[48] The effects could be attributed primarily to the reduction in distress in the immediate postoperative period that could have buffered the effects of stress hormones, facilitated recruitment of inflammatory cells at the wound site and reduced the rate of infections and the sustained elevated levels of proinflammatory cytokines at a later period. Secondly, various yogic breathing practices are known to increase oxygen consumption[49–51] that could hasten wound repair.[52] Lastly, our intervention also helped facilitate compliance to treatment such as the use of breathing exercises that are known to reduce risks of pulmonary infections following surgery.[35] We compared our intervention with standard physiotherapy rehabilitation and supportive therapy that has earlier shown beneficial effects with respect to postoperative and wound healing outcomes.[53] This study supports the hypotheses that adding an active stress reduction component (yoga-based relaxation and breathing exercises) to these interventions would hasten the recovery process.

Our study however, has several limitations: i) We assessed only one cytokine TNF-alpha in our study; other cytokines and biological determinants (IL-1, IL-10, transforming growth factor beta (TGF β), fibroblast growth factor (FGF), vascular endothelial growth factor (VEGF)) that are known to impact wound healing at various stages and intervals following surgery were not assessed.[36] ii) We assessed plasma TNF-alpha levels only after four weeks of surgery and not immediately afterwards; an immediate postoperative TNF-alpha level would have helped determine if changes were occurring due to perioperative or postoperative factors. Also, assessing these levels immediately following surgery and after subsequent intervals thereafter, would have provided more valuable information on the dynamics of wound healing. iii) The results of this study are also limited by the fact that there was 29% attrition in the study on follow-up assessments, further affecting the power of our study. iv) Differences in the preoperative nutritional status of the subjects could have confounded the observations in this study. However, it may be pointed out that although a detailed nutritional assessment of each subject was not done, all subjects were evaluated for any clinical signs of malnutrition and hemoglobin levels before surgery. All subjects were moderately built and did not show any clinical signs of malnutrition or abnormal hemoglobin levels. We did not assess serum albumin levels as none of the subjects showed any clinical signs of malnutrition. This measure could have been used to assess differences in nutritional status across subjects.

However, our results support the beneficial effects of yoga intervention on both postsurgery outcomes and wound healing. Further studies on the dynamics of wound repair processes using more relevant and advanced biological determinants of stress and wound repair are warranted. Future studies should evaluate the mechanisms of action of yoga intervention through possible neuroendocrine and immune markers.
